# Virtual non-contrast series of photon-counting detector computed tomography angiography for aortic valve calcium scoring

**DOI:** 10.1007/s10554-023-03040-4

**Published:** 2024-01-04

**Authors:** Franka Risch, Eva Harmel, Katharina Rippel, Bastian Wein, Philip Raake, Evaldas Girdauskas, Sébastien Elvinger, Tamer Owais, Christian Scheurig-Muenkler, Thomas Kroencke, Florian Schwarz, Franziska Braun, Josua A. Decker

**Affiliations:** 1 Department of Diagnostic and Interventional Radiology and Neuroradiology, University Hospital Augsburg, Stenglinstr. 2, 86156 Augsburg, Germany; 2 Medical Clinic, Department of Cardiology, University Hospital Augsburg, Augsburg, Germany; 3 Department of Cardiac Surgery, University Hospital Augsburg, Augsburg, Germany; 4 Centre for Advanced Analytics and Predictive Sciences, Augsburg University, Augsburg, Germany; 5 Clinic for Diagnostic and Interventional Radiology, Donau-Isar-Klinikum, Deggendorf, Germany

**Keywords:** Photon-counting detector computed tomography, Aortic valve calcium quantification, Virtual non-contrast, Calcium-sensitive algorithm, Radiation dose reduction potential

## Abstract

The aim of our study was to evaluate two different virtual non-contrast (VNC) algorithms applied to photon counting detector (PCD)-CT data in terms of noise, effectiveness of contrast media subtraction and aortic valve calcium (AVC) scoring compared to reference true non-contrast (TNC)-based results. Consecutive patients underwent TAVR planning examination comprising a TNC scan, followed by a CTA of the heart. VNC series were reconstructed using a conventional (VNC_conv_) and a calcium-preserving (VNC_pc_) algorithm. Noise was analyzed by means of the standard deviation of CT-values within the left ventricle. To assess the effectiveness of contrast media removal, heart volumes were segmented and the proportion of their histograms > 130HU was taken. AVC was measured by Agatston and volume score. 41 patients were included. Comparable noise levels to TNC were achieved with all VNC reconstructions. Contrast media was effectively virtually removed (proportions > 130HU from 81% to < 1%). Median calcium scores derived from VNC_conv_ underestimated TNC-based scores (up to 74%). Results with smallest absolute difference to TNC were obtained with VNC_pc_ reconstructions (0.4 mm, Br36, QIR 4), but with persistent significant underestimation (median 29%). Both VNC algorithms showed near-perfect (r²>0.9) correlation with TNC. Thin-slice VNC reconstructions provide equivalent noise levels to standard thick-slice TNC series and effective virtual removal of iodinated contrast. AVC scoring was feasible on both VNC series, showing near-perfect correlation, but with significant underestimation. VNC_pc_ with 0.4 mm slices and Br36 kernel at QIR 4 gave the most comparable results and, with further advances, could be a promising replacement for additional TNC.

## Introduction

The prognostic value of aortic valve calcium (AVC) in patients prior to TAVR is well established [1–5]. In 2021, the European Society of Cardiology/European Association for Cardio-Thoracic Surgery guidelines for the management of valvular heart disease further emphasized the importance of AVC scoring on cardiac computed tomography (CCT) images for class IIa indications prior to aortic valve replacement. To assess the feasibility of a TAVR procedure by evaluating anatomical details of the aortic valve and vascular access, as well as to calculate annular dimensions, CT angiography (CTA) is considered the gold standard in the diagnostic workup [6, 7]. However, accurate quantification of AVC in TAVR patients requires an additional true non-contrast scan (TNC), which naturally increases radiation exposure to the patient.

CT systems capable of acquiring spectral data allow virtual subtraction of iodine contrast from CTA series. The resulting virtual non-contrast (VNC) series promises to eliminate the need for separate TNC series, reducing patient’s radiation dose and acquisition time [8, 9]. In addition to the well-known techniques, such as dual-energy, kV-switching or dual-layer based on energy-integrating detectors, novel photon-counting detectors inherently provide spectral information for each scan performed. Recent studies have demonstrated the reliability of VNC-measured coronary calcium scores, with excellent correlation to TNC-measured scores [10–14]. Because calcium and iodine have similar attenuation characteristics, conventional VNC algorithms (VNC_conv_) partially subtract the calcium contrast, resulting in an underestimation of the score. A novel calcium-preserving VNC algorithm (PureCalcium, VNC_pc_) performs additional steps to differentiate between iodine and calcium prior to contrast subtraction and restores calcium contrast subsequently [11].

The study objective was to evaluate the feasibility of AVC quantification on both conventional and calcium-preserving VNC algorithms, derived from PCD CTA datasets compared to reference TNC values. Furthermore, we investigated the influence of different reconstruction settings on noise, effectiveness of iodine removal and tested the acquired VNC calcium scores for their predictive accuracy compared to TNC.

## Materials and methods

This retrospective single-center study at the University Hospital Augsburg was approved by the institutional review board with a waiver for written informed consent. The trial was reviewed and cleared by local ethics committee of the Ludwig Maximilian University of Munich (project number 22-0456).

### Study population

Consecutive patients who followed the institution’s standard pre-TAVR scanning protocol between June and September 2021 were included in the study cohort and allowed for further processing and analysis of CT images (n = 45). Patients with status post aortic valve replacement or non-measurable massive calcification, e.g., severe calcification including the aortomitral continuity, were excluded from the analysis (n = 4).

### Data acquisition

All scans were performed on a first generation, dual-source PCD-CT (NAEOTOM Alpha, Siemens Healthineers, Erlangen, Germany). The scan protocol included two contrast phases, first, a pre-contrast acquisition of the heart (true non-contrast, TNC) and second a CTA of the heart, aorta, and iliac arteries. Both scans were performed with a high pitch of 3.2 and ECG-triggered. The tube voltage was 120 kVp and the detector collimation 144 × 0.4 mm². By setting the image quality level to 19 and 64 for TNC and CTA, respectively, the reference tube current time product was adjusted. For the CTA a triphasic contrast injection protocol with bolus tracking was used, following institutional standard. In the first phase 60 ml of undiluted contrast material (Iopromide Ultravist 300 mgI/ml, Bayer Vital, Leverkusen, Germany) was injected followed by a mixture of 30 ml contrast material and 30 ml 0.9% saline solution and chased with 20 ml 0.9% saline solution. A flow of 5 ml/s was used in all three phases.

### Image reconstruction

TNC from pre-contrast and VNC_conv_ series from CTA raw data were directly reconstructed on the scanner console. VNC_pc_ reconstructions were performed on a dedicated workstation (ReconCT, Version 15.0, Siemens Healthineers, Erlangen, Germany), both using the best diastole. Only one TNC series was reconstructed as ground truth with standard settings, using a quantitative regular kernel (Qr36), virtual monoenergetic level of 70 keV, the quantum iterative reconstruction (QIR) off and slice thickness and increment of 3.0 and 1.5 mm. For reconstructions based on CTA, the approach of thin slices/increments (0.4/0.2 mm and 1.0/0.4 mm) was followed, as they were expected to reveal even very small calcifications and to max out the resolution capabilities of the CT detectors. To compensate for an increase in image noise, the iterative reconstruction levels were increased (Q3 and Q4). In addition to the proposed quantitative kernel (Qr36), a body kernel (Br36) was also used. All settings were combined with both VNC algorithms (conventional and PureCalcium at virtual monenergetic level of 70 keV). A detailed description of the settings resulting in four reconstructions for each VNC algorithm is given in Table 1.

**Table 1:** Image reconstruction settings for true non-contrast and virtual non-contrast, both conventional and pure calcium series

Series	Kernel	QIR level	Slice thickness [mm]	Slice increment [mm]
TNC	Qr36	off	3.0	1.5
VNC_conv_^1^/VNC_pc_^1^	Qr36	Q4	0.4	0.2
VNC_conv_^2^/VNC_pc_^2^	Br36	Q4	0.4	0.2
VNC_conv_^3^/VNC_pc_^3^	Qr36	Q4	1.0	0.4
VNC_conv_^4^/VNC_pc_^4^	Qr36	Q3	1.0	0.4

QIR = quantum iterative reconstruction, TNC = true non-contrast, VNC = virtual non-contrast (conv = conventional, pc = pure calcium)

The field of view was set for all series equivalently to 180 × 180 mm², covering the whole heart. The main difference between the VNC algorithms used, is in the handling of calcium. Since the attenuation properties of iodinated contrast media and calcium are similar, subtraction of iodine alone will also result in partial removal of the calcium component as in VNC_conv_. By differentiating between iodine and calcium prior to the iodine subtraction step, the VNC_pc_ algorithm reconstructs the calcium contrast afterwards.

### Image analysis

Image analysis was divided into three parts including noise analysis, effective iodine subtraction assessment and AVC quantification. Noise was measured by positioning a 15 mm diameter region of interest (ROI) within the left ventricle on three different slices of the CTA series using commercial imaging software (DeepUnity, Dedalus HealthCare, Bonn, Germany). ROIs were automatically transferred to the TNC and VNC reconstructions and the mean and standard deviation of the CT values were recorded. The standard deviation averaged over the three slice positions was used as a measure of image noise. To assess effective virtual iodine subtraction, for each patient the series were transformed to obtain isotropic 1 mm³ voxels, registered and a semi-manual segmentation of the whole heart was performed using open-source software (Slicer3D, www.slicer.org). CT value distributions were compared between CTA, TNC and VNC series. VNC_conv_^1^ and VNC_pc_^1^ reconstructions were used as representative for each algorithm. AVC quantities were measured semi-manually with a commercially available software (syngo.CT, CaScoring workflow, Siemens Healthineers, Erlangen, Germany) considering contiguous voxels with a CT value above a threshold of 130 HUs. Both, Agatston and Volume score of the aortic valve were analyzed for all, TNC and VNC series.

### Statistical analysis

Statistical analyses were performed using python (version 3.8.1). The Shapiro-Wilk test was applied to assess value distribution. The paired t-test and the Wilcoxon signed-rank test were used to test for differences in parametric and non-parametric data respectively. For all linear regression related presentations and calculations, data were square root transformed prior to analyses to improve homoscedasticity. To obtain the predictive accuracy of calcium quantities in virtual-unenhanced series, a 10,000-fold bootstrap was performed on a linear regression model. The mean absolute error was calculated as the absolute difference between the predicted, back-transformed and the TNC measured calcium quantity. Binary data are presented in frequencies (proportions) and continuous data with mean ± standard deviation or as median with interquartile range, as individually indicated. P-values < 0.05 were considered to indicate statistical significance.

## Results

### Baseline study characteristics

**Fig. 1:**
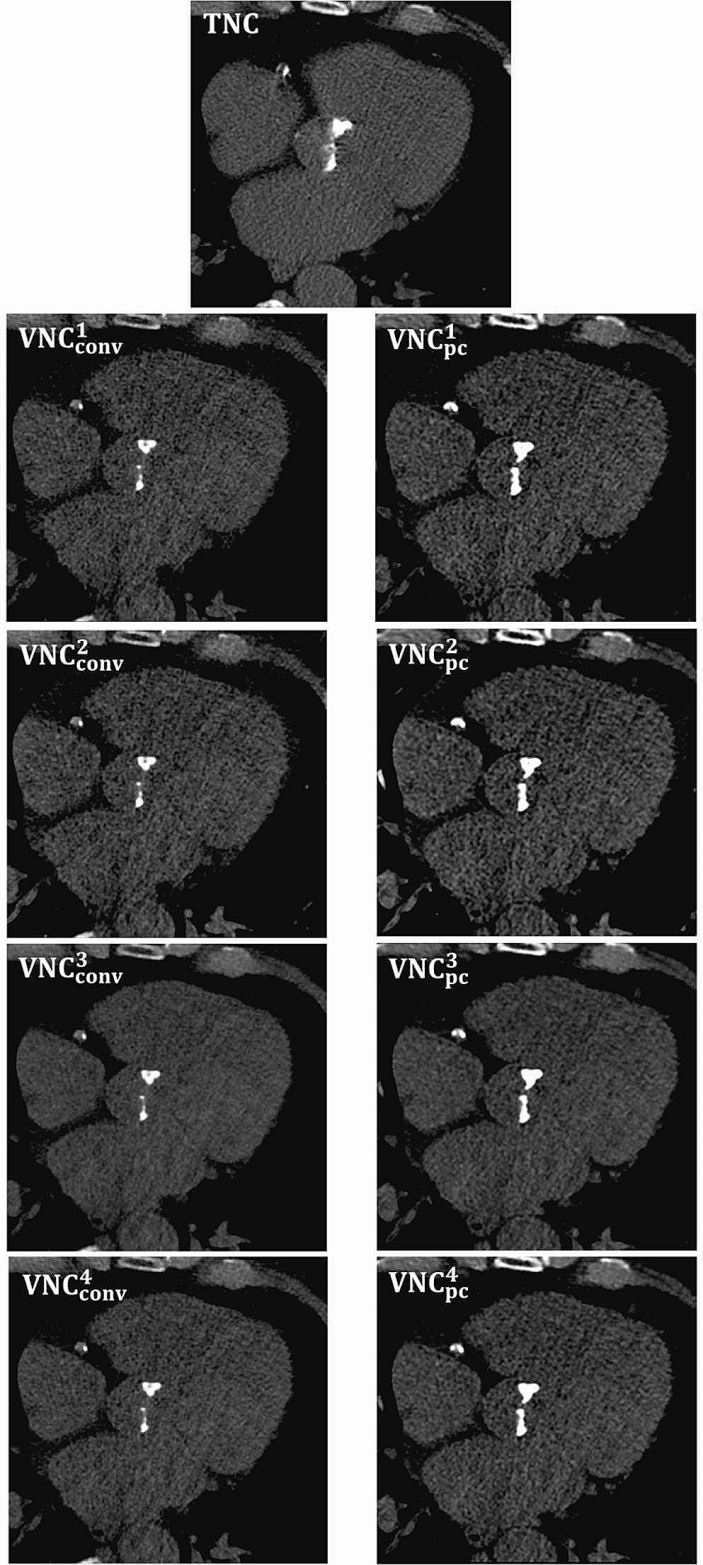
Demonstration of the aortic calcification in an axial slice for true non-contrast (TNC), conventional (VNC_conv_) and pure calcium virtual non-contrast (VNC_pc_) series for all VNC^x^ reconstruction settings (x = 1–4)

A total of 45 patients were primarily enrolled. Four patients were excluded due to status post aortic valve replacement (n = 3) and non-segmentable massive calcification of the aortic valve and aortomitral continuity (n = 1) according to the exclusion criteria. The other patients (n = 41), thereof 17 (41.5%) women, were included in the study. Regarding CT radiation dose, median volumetric CT dose index (CTDI_vol_) and dose length product (DLP) were 1.5 (1.2–1.9) mGy and 31.8 (23.5–38.8) mGy*cm for the pre-contrast, and 4.4 (3.6–5.2) mGy and 330.0 (270–410) mGy*cm for CTA scans, respectively. Mean AVC on TNC series was 2829 ± 1618 and 2242 ± 1253 mm^3^ for Agatston and volume score, respectively. Further baseline characteristics of the study cohort are shown in Table 2. Figure 1 visualizes all reconstructions considered at the same axial slice position. All results of the evaluations performed for noise, virtual iodine subtraction and aortic valve calcification are summarized in Table 3.

**Table 2:** Baseline study characteristics

Total n = 41		
**Clinical**		
Age [years]	80.0 (75.0–83.0)	
Male	24 (58.5%)	
BMI [kg/m²]	25.9 (23.6–32.4)	
Aortic Valve Area [cm²]	0.72 ± 0.22	
**Cardiovascular risk factors**		
Arterial hypertension	30 (73.2%)	
Current or former smoker	10 (24.4%)	
Diabetes	18 (43.9%)	
Hypercholesterolemia	15 (36.6%)	
Positive family history for adverse cardiovascular events	1 (2.4%)	
Obesity	11 (26.8%)	
**CT radiation dose**	**Pre-contrast**	**CTA**
CTDI_vol_ [mGy]	1.5 (1.2–1.9)	4.4 (3.6–5.2)
DLP [mGy*cm]	31.8 (23.5–38.8)	330.0 (270–410)
Effective mAs [mAs]	21 (17–26)	62 (52–77)
SSDE [mGy]	2.0 (1.7–2.2)	5.4 (4.8–6.1)
**Coronary artery calcium**		
TNC Agatston Score	2829 ± 1618	
TNC Volume Score [mm³]	2242 ± 1253	

Values are mean ± standard deviation, median (interquartile range), or frequency (percentage). BMI = body mass index, CTDI_vol_ = computed tomography dose index, DLP = dose length product, SSDE = size specific dose estimate, TNC = true non-contrast

**Table 3:** Summarized results of the evaluations performed for noise, virtual iodine subtraction and aortic valve calcification

	Noise		Virtual iodine subtraction		Aortic valve calcification				
	[HU]	p-value	Histogram > 130 HU	p-value	Absolute values Agatston score Volume score [mm³]	Percentage difference In Agatston score In Volume score	p-value	r²	MAE
TNC	27 ± 4		0.5 (0.4–1.2) %		2800 (2075–3682)				
					2206 (1645–2895)				
VNC_conv_^1^	28 ± 5	p < .001	0.2 (0.1–0.5) %	p < .001	752 (390–1357)	−69 ± 11%	p < .001	0.91	405 (351–475)
					653 (320–1052)	−69 ± 11%	p < .001	0.91	316 (276–369)
VNC_pc_^1^	33 ± 5	p < .001	0.6 (0.4–1.3) %	p = 1.0	1986 (1270–3278)	−25 ± 20%	p < .001	0.91	448 (388–520)
					1515 (971–2480)	−28 ± 20%	p < .001	0.91	357 (308–416)
VNC_conv_^2^	29 ± 5	p < .001			777 (408–1381)	−69 ± 11%	p < .001	0.92	378 (328–441)
					652 (333–1064)	−69 ± 11%	p < .001	0.92	296 (257–344)
VNC_pc_^2^	33 ± 5	p < .001			2023 (1320–3282)	−25 ± 19%	p < .001	0.92	426 (368–493)
					1536 (1009–2479)	−28 ± 19%	p < .001	0.91	341 (294–396)
VNC_conv_^3^	19 ± 4	p < .001			731 (383–1279)	−71 ± 11%	p < .001	0.91	404 (348–478)
					591 (316–980)	−71 ± 11%	p < .001	0.92	308 (267–363)
VNC_pc_^3^	22 ± 4	p < .001			1760 (1180–3082)	−32 ± 19%	p < .001	0.93	392 (339–453)
					1351 (900–2341)	−35 ± 19%	p < .001	0.93	305 (263–353)
VNC_conv_^4^	23 ± 4	p < .001			757 (391–1302)	−70 ± 11%	p < .001	0.92	393 (338–468)
					610 (323–1022)	−70 ± 11%	p < .001	0.92	301 (261–356)
VNC_pc_^4^	27 ± 4	p = 1.0			1808 (1183–3121)	−31 ± 19%	p < .001	0.93	394 (339–456)
					1396 (907–2374)	−34 ± 19%	p < .001	0.92	309 (265–358)

Values are mean ± standard deviation or median (interquartile range); MAE = mean absolute error, TNC = true non-contrast., VNC = virtual non-contrast (conv = conventional, pc = pure calcium). P-values refer to comparison with ground truth (TNC) and are corrected using the Bonferroni method

### Image noise

**Fig. 2:**
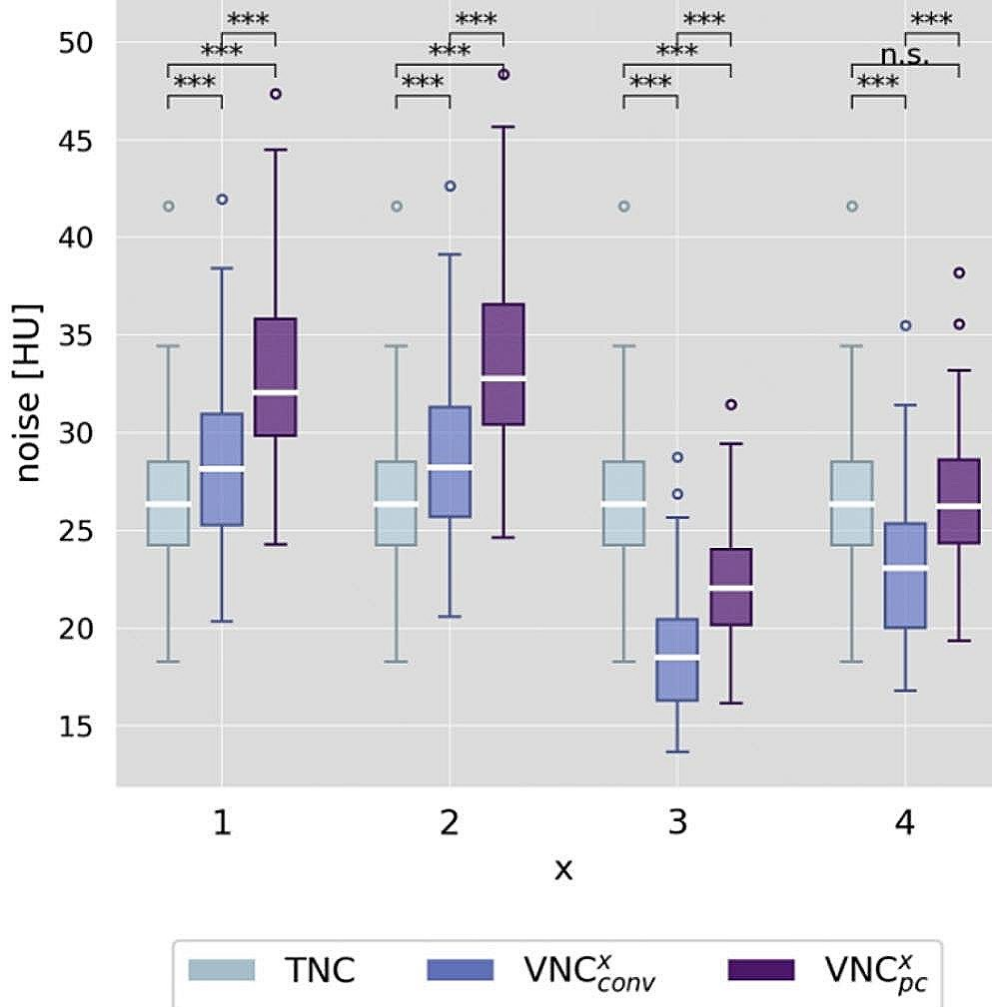
Boxplot of measured image noise. Noise is assessed as ROI’s (region of interest) in the left ventricular cavity and compared between true non-contrast (TNC) and virtual non-contrast (conventional VNC_conv_ and pure calcium VNC_pc_) series for each different reconstruction setting of VNC^x^ (x = 1–4). Stars mark significant differences as * = p < .05, ** = p < .01, *** = p < .001 and n.s. marks no significant difference

Image noise levels, assessed as standard deviation of CT values in ROIs in the left ventricular cavity, showed significant differences between TNC (27 ± 4 HU) and all VNC_conv_ and most VNC_pc_ series (p < .001) as demonstrated in the boxplot in Fig. 2. For reconstruction settings x = 1, 2 noise on VNC was on average higher (VNC_conv_^1,2^ = 28 ± 5, 29 ± 5 HU and VNC_pc_^1,2^ = 33 ± 5, 33 ± 5 HU) and for x = 3, 4 noise was lower compared to TNC (VNC_conv_^3,4^ = 19 ± 4, 23 ± 4 HU and VNC_pc_^3^ = 22 ± 4 HU). However, differences were small (on average < 6 HU). Only VNC_pc_^4^ showed no significant difference in noise level (VNC_pc_^4^ = 27 ± 4 HU).

### Virtual iodine subtraction

**Fig. 3:**
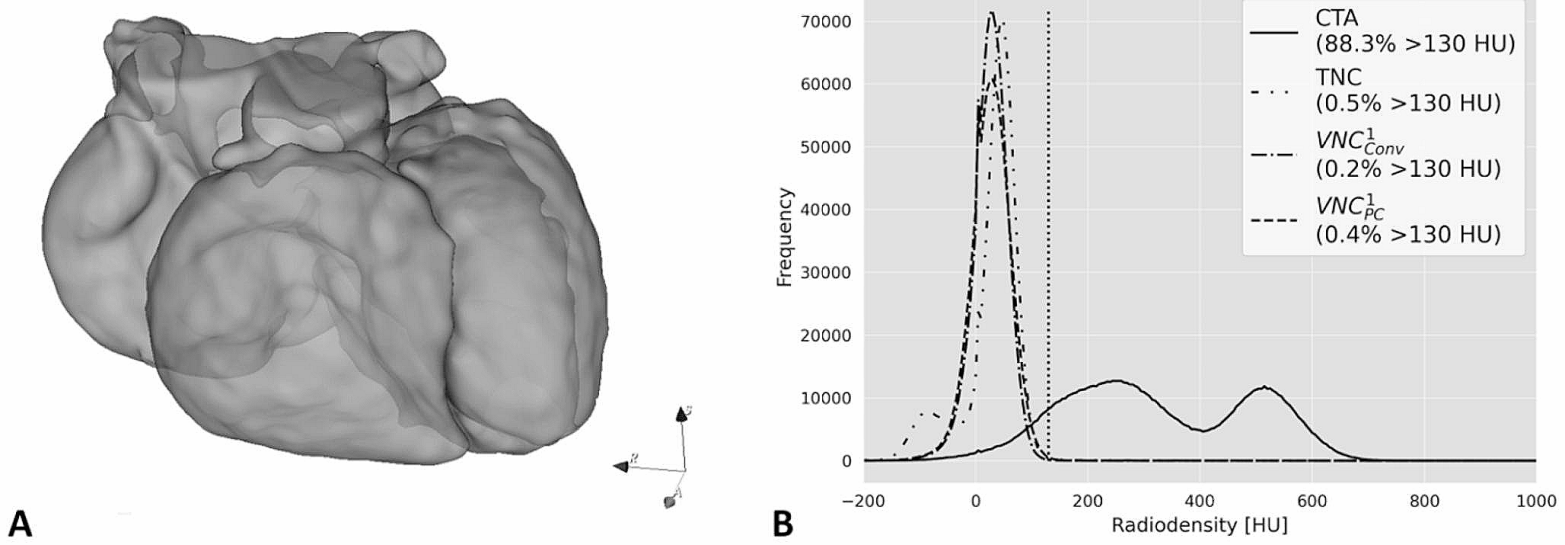
Effectiveness of iodine removal. **A** Demonstrates the segmentation of the whole heart and **B** shows the histograms based on the respective reconstruction (CTA = CT angiography, TNC = true non-contrast, VNC_conv_^1^ = conventional virtual non-contrast, and VNC_pc_^1^ = pure calcium virtual non-contrast). The histogram proportion exceeding 130 HU (marked by the dotted line) is given in the legend

Figure 3 shows the principle of how the effectiveness of virtual iodine subtraction was measured. The CT value histograms of the whole heart volume were compared between CTA, TNC, conventional and pure calcium VNC. As representatives only reconstructions x = 1 were used. Median proportions exceeding 130 HU were 81%, 0.5%, 0.2% and 0.6% for CTA, TNC, VNC_conv_^1^ and VNC_pc_^1^, respectively. Differences were significant between CTA and all non-contrast series (p < .001). The proportions of TNC greater than 130 HU were also significantly different from VNC_conv_^1^ (p < .001) but not from VNC_pc_^1^ (p = 1.0).

### Calcium quantification

**Fig. 4:**
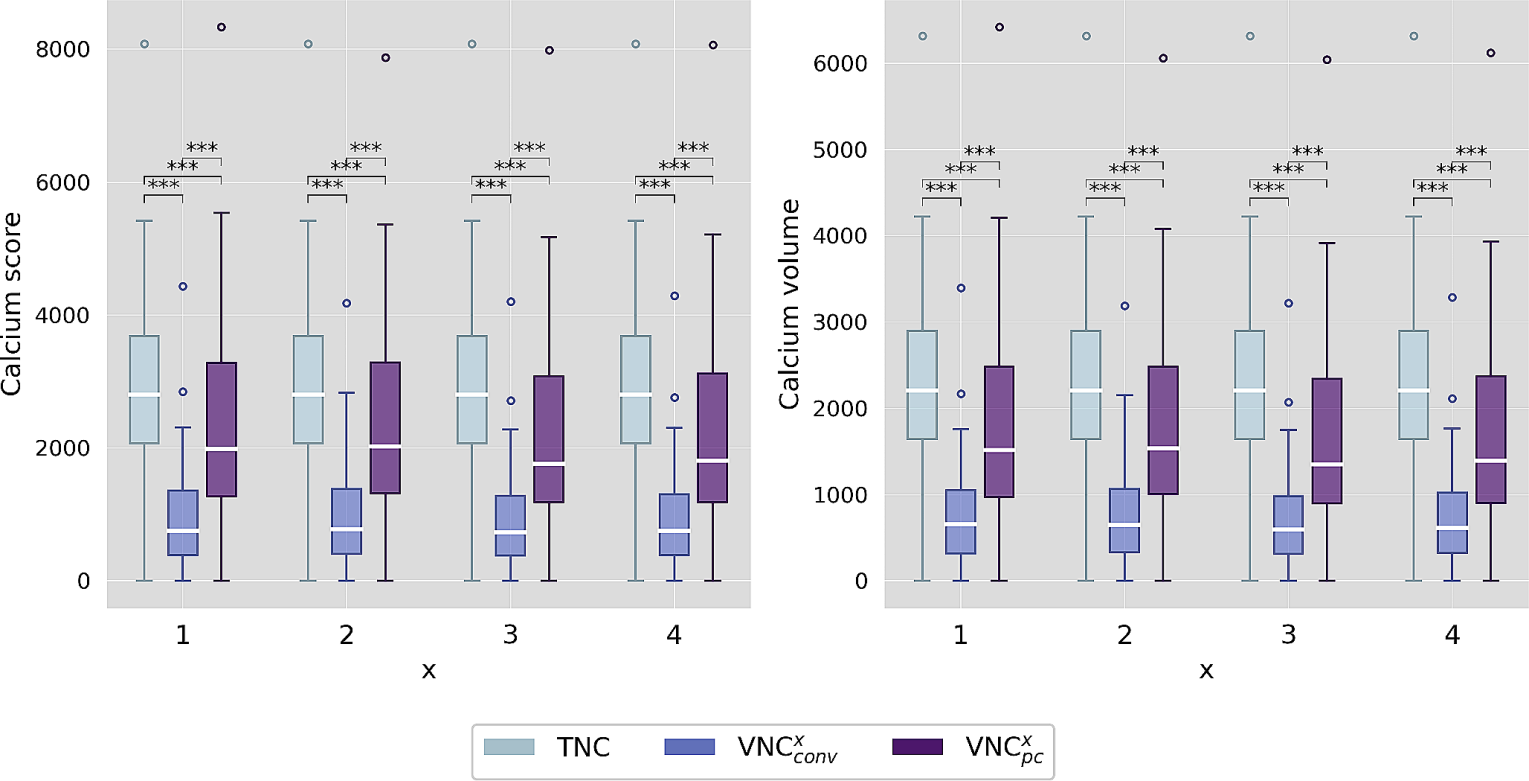
Boxplot of measured calcium quantities comparing true non-contrast (TNC) and virtual non-contrast (conventional VNC_conv_ and pure calcium VNC_pc_) series for each different reconstruction setting of VNC^x^ (x = 1–4). Stars mark significant differences as * = p < .05, ** = p < .01, *** = p < .001 and n.s. marks no significant difference

**Fig. 5:**
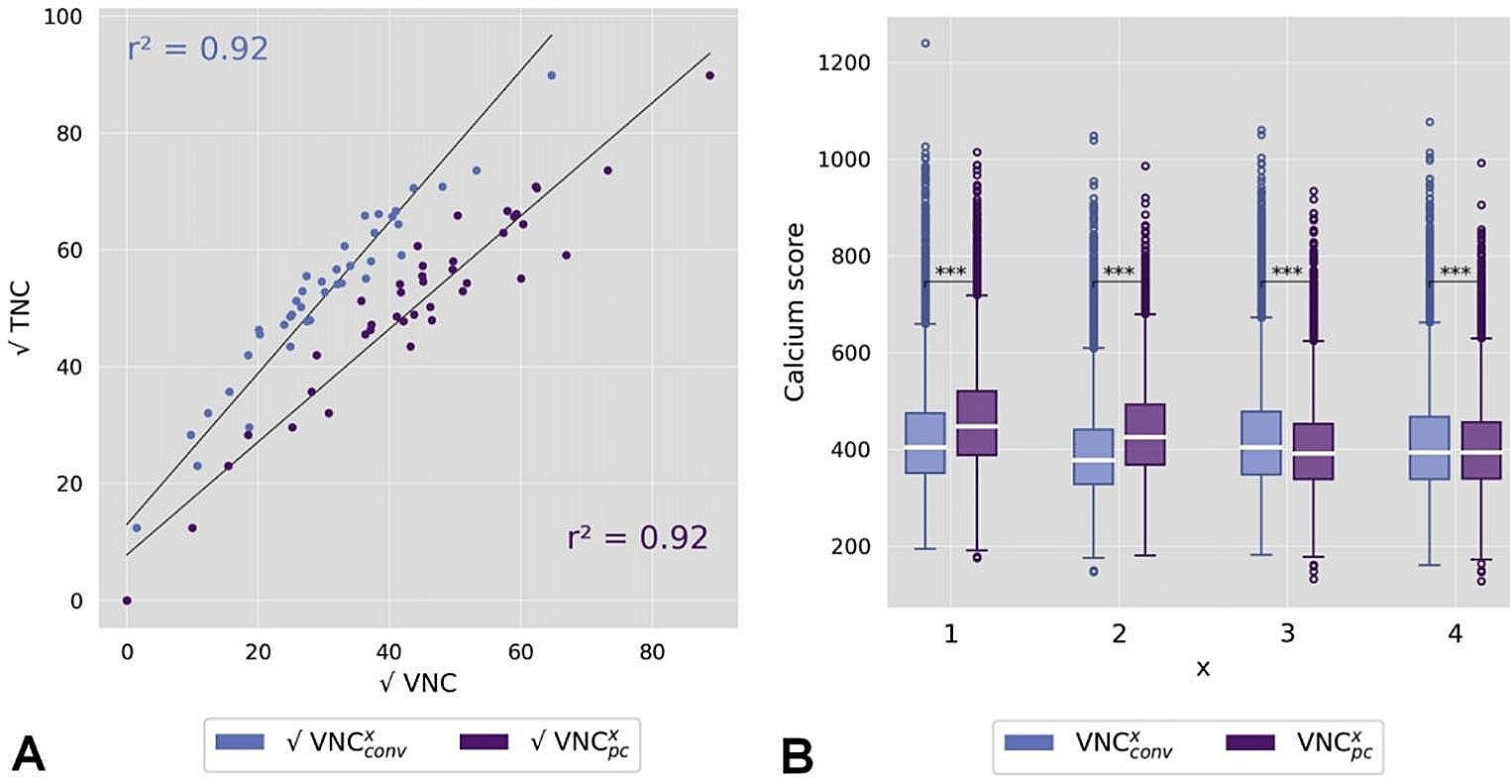
**A** Shows the linear regression analyses of square root transformed Agatston scores derived from true non-contrast (TNC) vs. virtual non-contrast (VNC, conv = conventional and pc = pure calcium) for reconstruction setting x = 2. r² = coefficient of determination. **B** Shows the calculated mean absolute error from 10,000-fold bootstrapping analysis for the Agatston score and all reconstruction settings of VNC^x^ (x = 1–4)

Median calcium quantities were 2800 and 2206 mm³ on TNC. Mean percentage differences of VNC_conv_^1–4^ to TNC were − 69%, −69%, −71%, −70% and − 69%, −69%, −71%, −70%and of VNC_pc_^1–4^ to TNC-25%, −25%, −32%, −31% and − 28%, −28%, −35%, −34%, for score and volume respectively (Fig. 4). Measurements on all VNC reconstructions significantly underestimated calcium quantities with TNC as ground truth (p < .001). However, the underestimation in the score was more than twice as high for VNC_conv_ compared to VNC_pc_. The differences between the individual reconstruction settings x = 1–4 were relatively small, but x = 2 (body kernel, maximum iteration, super thin slices) gave the best results with the smallest average underestimation. Two patients had no AVC and an equivalent score of zero for TNC and all VNC reconstructions. Linear correlation of TNC and all VNC based calcium quantities was excellent (all r² > 0.9) without striking differences between the two VNC algorithms or reconstruction settings. In Fig. 5 a linear regression is demonstrated for Agatston scores of series x = 2. However, the results of the bootstrap analysis showed a small trend towards higher absolute mean errors in predicting calcium scores based on a linear regression model for VNC_pc_ compared to VNC_conv_. The median error was 405, 378, 404, 398 and 316, 296, 308, 301 mm³ for VNC_conv_^1–4^ and 448, 426, 392, 394 and 357, 341, 305, 309 mm³ for VNC_pc_^1–4^ for score and volume, respectively. Figure 5B shows the results for the Agatston score and all series x = 1–4.

## Discussion

This study evaluated the performance of different VNC algorithms and reconstruction parameters for virtual AVC scoring on PCD-CT CTA series. The main findings of this study are: (1) Iodine contrast was effectively removed in all VNC series; (2) AVC values were significantly underestimated on all VNC reconstructions, with little effect of the reconstruction setting, however, differences to TNC-based values were more than twice as large for VNC_conv_ compared to VNC_pc_; (3) Although the linear correlation was excellent for all VNC to TNC based AVC measures, the prediction error was negligibly higher for VNC_pc_ reconstructions than for VNC_conv_ reconstructions.

In the spreading field of catheter-based procedures, aortic stenosis remains the most important indication for catheter-based valve replacement [15]. Various CT derived parameters, e.g., AVC quantity, play a tremendous role in procedure planning, patient selection and medical indication for treatment [7, 16]. The precise quantification of AVC in TAVR patients hitherto relies on a TNC scan, acquired prior to CTA for TAVR planning. Substituting the TNC scan with a VNC series derived from the CTA may reduce both radiation and examination time.

As been described for dual-energy CTAs, coronary artery calcium (CAC) quantities derived from PCD-CT series using the VNC_conv_ algorithm were approximately 50% lower but showed excellent linear correlation with TNC calcium quantities [10, 13, 17]. Thus, a correction factor can be applied to allow comparability with TNC series. A possible reason for the 70% discrepancy between TNC and VNC_conv_ in this study may be the extent of calcification. While CAC quantities are predominantly < 1000 in Agatston score, the interquartile range in this study was from 2000 up to 3700. The novel calcium-preserving VNC_pc_ algorithm promised to eliminate the additional transformation step by providing full calcium contrast, however, the study situation is sparse. CAC scores on VNC_pc_ derived from PCD CT showed a reduced underestimation of the ground truth results of approximately 26% in the median [11]. Our results are consistent with this, with calcium scores on VNC_pc_ reconstructions more than twice as high as those on VNC_conv_ reconstructions. However, scores were still significantly underestimated compared to TNC and no superiority of VNC_pc_ was observed in terms of linear correlation to TNC. The question is, how much variation in AVC score is acceptable? Most commonly, patients are divided into high and low AVC according to thresholds. Using an Agatston score of 1200 for women and 2000 for men [18], the best-rated reconstruction algorithm, VNC_pc_^2^, correctly classifies 14 out of 15 patients in women and 14 out of 18 in men into the high AVC group.

In contrast, VNC_conv_^2^ only correctly matches 4 and 2 for women and men. Even better results might be obtained by adjusting the monoenergetic level, which is possible for the VNC_pc_ algorithm. Recently, Fink et al. found that 60 keV VNC_pc_ to best match TNC results regarding CAC [14] and Mergen et al. additionally applied high iterative reconstruction levels and showed that 80 keV VNC_pc_ combined with QIR level 4 works best for AVC [19].

In the context of TAVR planning and also follow-up, PCD CT was already described as a promising technique [20] that could be further enhanced by using the inherent spectral data for calcium quantification. As radiation reduction and time efficiency play an important role in modern CT diagnostics, PCD-based VNC reconstructions are an alternative with the potential to replace dedicated TNC studies for AVC quantification.

Besides its retrospective design and being conducted on a single center, this study has several limitations. First, our cohort includes a rather small sample size, which seems justified by the extensive reconstructions using several parameters and the quantitative analyses of all series. Second, further studies are needed to confirm our findings and to assess the impact on related clinical decisions. Third, the choice of reconstruction settings should be extended to include different virtual monenergetic levels, as they seem to significantly affect the performance of AVC quantification on VNC_pc_. Finally, this study focuses only on quantitative parameters. A subjective evaluation and comparison of the reconstructed series could add more comprehensive information.

In conclusion this study proofed the feasibility of AVC scoring on VNC reconstructions derived from PCD CTA. Comparable noise levels and an effective virtual removal of iodinated contrast media could be demonstrated. In contrast to VNC_conv_, the novel calcium-sensitive VNC_pc_ algorithm provides an improved calcium contrast and more comparable AVC scores to TNC, but with a persisting significant underestimation. With further algorithmic advances, VNC_pc_ promises to be an adequate replacement for an additional TNC scan, minimizing radiation dose and examination time.

## Abbreviations

AVC: Aortic Valve Calcium
CT: Computed Tomography
CTA: Computed Tomography Angiography
PCD: Photon-Counting Detector
QIR: Quantum Iterative Reconstruction
TAVR: Transcatheter Aortic Valve Replacement
TNC: True Non-Contrast
VNC: Virtual Non-Contrast (conv = conventional, pc = pure calcium)
